# Complete Genome Sequence of a Western Siberian *Lymantria dispar* Multiple Nucleopolyhedrovirus Isolate

**DOI:** 10.1128/genomeA.00335-15

**Published:** 2015-04-23

**Authors:** Marsel R. Kabilov, Vyacheslav V. Martemyanov, Alexey E. Tupikin, Olga A. Baturina, Irina A. Belousova, Alexander A. Bondar, Alexandr V. Ilyinykh

**Affiliations:** a SB RAS Genomics Core Facility, Institute of Chemical Biology and Fundamental Medicine SB RAS, Novosibirsk, Russia; b Institute of Systematics and Ecology of Animals SB RAS, Novosibirsk, Russia

## Abstract

A novel strain of *Lymantria dispar* multiple nucleopolyhedrovirus (LdMNPV-27) was isolated from dead larvae of a Western Siberian (WS) population of gypsy moths (*Lymantria dispar* L.). We report the complete genome sequence of this strain, comprising 164,108 bp and double-stranded circular DNA encoding 162 predicted open reading frames.

## GENOME ANNOUNCEMENT

The nuclear polyhedrosis viruses (NPV), which belong to the genus *Alphabaculovirus*, are pathogenic for invertebrates, particularly insects of the order *Lepidoptera*. *Lymantria dispar* multiple NPV is the lethal viral infection of gypsy moths that causes epizootics in wild host populations. The gypsy moth (*Lymantria dispar* L.) is a widespread forest defoliator species inhabiting Europe, Asia, North America, and the northern part of Africa. There is no strong food specialization for the gypsy moth, so this herbivore species is one of the most important forest pests in the world.

Here, we present a complete genome for a novel strain of *Lymantria dispar* multiple nucleopolyhedrovirus (LdMNPV-27), isolated from dead larvae of a Western Siberian (WS) population of gypsy moths. The LdMNPV-27 natural isolate was passed through the larvae of a wild WS population of gypsy moths under laboratory conditions in 2006. Virus DNA was phenol-chloroform extracted from purified viral polyhedral inclusion bodies (1). The virus genomic DNA library prepared by a Nextera kit (Illumina) was analyzed using a MiSeq genome sequencer (2 × 300 cycles; Illumina) in the SB RAS Genomics Core Facility (ICBFM SB RAS, Novosibirsk, Russia). The full-length genome was assembled *de novo* with CLC GW version 7.0 software (CLC Bio).

The LdMNPV-27 strain has a double-stranded circular 164,108-bp DNA genome with an average 57.4% GC content. Potential open reading frames (ORFs) were identified by using the GeneMarkHMM (2) and ZCURVE_V software packages (3) and subsequently analyzed manually. Putative functions of the ORFs were predicted with the help of BLAST (NCBI) (4) and Blast2GO (5).

At least 162 ORFs of the total 174 predicted for the virus genome seem to be functional based on a genome comparative analysis with other known NPV genomes. The closest relative is LdMNPV strain 3029 (KM386655) (6) with 168 ORFs. The average similarities between these viruses at the DNA (CDS) and protein levels are 96% and 99%, respectively. We conclude that LdMNPV-27 can be related to the Asian group of the nuclear polyhedrosis viruses.

### Nucleotide sequence accession number. 

The genome sequence of LdMNPV-27 has been deposited in GenBank under the accession number KP027546.
